# Direct Cost of Parkinson's Disease: A Real-World Data Study of Second-Line Therapies

**DOI:** 10.1155/2020/9106026

**Published:** 2020-05-12

**Authors:** Elisa Gomez-Inhiesto, María Teresa Acaiturri-Ayesta, Iker Ustarroz-Aguirre, Diana Camahuali, Maider Urtaran-Laresgoiti, Marisol Basabe-Aldecoa, Roberto Nuño-Solinís, Elena Urizar

**Affiliations:** ^1^Dirección Económico-Financiera, Hospital Universitario Cruces—OSI Ezkerraldea Enkarterri Cruces, Barakaldo, Biscay, Spain; ^2^Deusto Business School Health, University of Deusto, Bilbao, Spain

## Abstract

Parkinson's disease is one of the main reasons for neurological consultation in Spain. Due to the nature of the disease, it impacts patients, families, and caregivers. Parkinson's disease is a degenerative disease with no cure, although second-line therapies have recently improved the quality of life of patients in advanced stages. The aim of this study was to analyse the costs of the following therapies: deep brain stimulation (DBS), continuous duodenal levodopa/carbidopa infusion (CDLCI), and continuous subcutaneous apomorphine infusion (CSAI). The methodology used was based on real-world data obtained from an integrated healthcare organization in the Basque Country from 2016 to 2018. This bottom-up retrospective approach only took into account the healthcare perspective. The results revealed the annual cost over 3 years and the projected cost for an additional 2 years. The total costs for 5 years of treatment were as follows: €53,217 for DBS, €208,163 for CDLCI, and €170,591 for CSAI. These costs are in line with those found in the available literature on the subject. Additionally, the analysis provided details of the different costs incurred during intervention with the therapies and compared the costs to those reported in other studies.

## 1. Introduction

Parkinson's disease (PD) is the second most common degenerative disease of the nervous system after Alzheimer's disease. The incidence is 21/100,000; the disease mainly affects older adults, and its prevalence increases with age. In Spain, the incidence is 1.9/1,000, making it the second most common cause of neurological consultation. Due to the nature of the disease, it is considered to be underdiagnosed [1–3].

PD can be associated with nonmodifiable risk factors such as age and gender. In men, the prevalence and incidence can be 1.5 to 2 times higher, respectively, than those in women. Other risk factors include family history and exposure to agricultural pesticides and some drugs, such as lithium and antipsychotics, while genetic risk factors have also been described [3, 4].

The initiation and development of PD and most of the motor symptoms of the disease are due to the progressive loss of substantia nigra (SN) neurons in the dopaminergic pathway. The most characteristic motor symptoms of PD, such as resting tremor, muscle stiffness, gait disturbances, and micrographia, are induced when the dopaminergic pathway is affected. Nonmotor symptoms may include pain, fatigue, sexual dysfunction, loss of autonomy, cognitive impairment, and psychiatric symptoms [1, 5–7].

Disease progression is gradual and slow. As there is no cure for PD, treatment is symptomatic and focuses on improving quality of life. In the early stages, therapeutic strategies involve orally administered drugs and physical and nutritional therapy. The second-line therapies used in advanced stages can be more invasive. These include deep brain stimulation (DBS), continuous duodenal levodopa/carbidopa infusion (CDLCI), and continuous subcutaneous apomorphine infusion (CSAI), which are the focus of this study. There is no consensus regarding the causes of advanced PD. However, many researchers agree that it is linked to refractory symptoms following conventional treatment and involves alternating periods when motor symptoms respond to therapeutics and periods when they do not. In addition, dyskinesia is moderate to severe in advanced PD and is accompanied by a loss of autonomy and cognitive disability.

As Parkinson's disease is a chronic, progressive, and irreversible condition, it has a great social impact. The quality of life of patients, families, and caregivers is extremely affected due to the increasing care required as the disease progresses and the resulting high expenses. In recent years, the survival rates of PD have improved due to population ageing, technological advances, and an improvement in social and health benefits in developed countries. Thus, patients require more years of care, and costs have consequently risen, presenting a challenge to the sustainability of the health system in the long term [2, 5, 6].

Reviews by Boloventa et al. [8] and Rodríguez-Blázquez et al. [9] summarized cost studies of the application of diverse methodologies for PD, but there have been very few studies based on real-world data (RWD). In this regard, the importance of this study lies in the source of the information and the possibility of using the results for better decision-making. Furthermore, the methodology may be able to be replicated as it uses reliable data from three therapies for the treatment of advanced PD.

## 2. Objective

The purpose of this study is to analyse the cost of the three main forms of treatment for Parkinson's disease through real-world data from an integrated healthcare organization in the Basque Country in Spain.

## 3. Methodology

The data for the analysis were obtained from the patient cost information system (PCIS) at the integrated health organization (IHO) known as Ezkerraldea Enkarterri Cruces. At this organization, a model of cost per patient was designed and implemented using a bottom-up methodology that connects all sources of information generated in clinical practice with economic information. This system provides individualized cost per patient in detail, allowing the traceability of all clinical care, including primary and specialist care. This information system allows for the analysis of costs based on diagnosis, patient, or procedure and includes all resources consumed (hospitalization, operating rooms, prostheses, pharmacy, consultations, laboratory, radiology, etc.). The PCIS enables the study of the variability of costs derived from clinical practice, as well as differences in consumption between patients.

**Figure 1:**
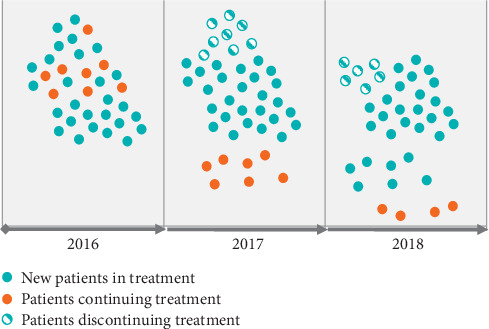
Transition of patients through treatment phases from 2016 to 2018.

This is a retrospective study that analysed direct costs from a healthcare perspective (not social costs). The data were from the period from 2016 to 2018 and from all patients with a diagnosis of PD who received one of the following three forms of treatment: deep brain stimulation (DBS), continuous duodenal levodopa/carbidopa infusion (CDLCI), or continuous subcutaneous apomorphine infusion (CSAI). In addition, all the healthcare costs for each patient, including hospital care, primary care, and outpatient pharmacy costs, were taken into account. Patients with PD go through different treatment phases. The PCIS enabled the identification of patients who started treatment, continuers, and patients who discontinued treatment in each year of the study. This approach allowed us to take into account the real costs of the analysis. Patient transition data were included for calculation purposes (Figure 1).

After determining the average annual cost of these forms of treatment, the information was extrapolated two years forward. This allows us to compare the full course of treatment, including the battery replacement required by the neurostimulator device in the fifth year of DBS.

## 4. Results

A total of 938 patients diagnosed with PD were treated from 2016 to 2018 and incurred a total cost of 11,003.856 euros at the IHO. A total of 105 patients from this initial group received one of the three types of treatment evaluated in this study; 57% of patients were treated with DBS, 25.7% with CDLCI, and 17.3% with CSAI.

### 4.1. Deep Brain Stimulation (DBS)

For DBS treatment, two specific times at which costs varied were identified. In the first year of treatment, physicians implanted electrodes in a specific area of the brain and the impulse generator device in the thorax. In patients treated at this organization, the impulse generator batteries were replaced 4.8 years following implantation. There was a total of 60 patients, and Table 1 shows the number of patients that underwent the implantation and replacement procedures.

During the first year of treatment, when the neurostimulator device was implanted, the average annual cost per patient was 32,363 euros (standard deviation of 5,067 euros). Fifty percent of this amount corresponded to the cost of the device (16,181.5 euros), 33% to the cost of the operating room (10,679.79 euros), 12% to the cost of hospitalization (3,883.56 euros), 3% to outpatient pharmaceutical products (970.89 euros), and 2% to in-hospital consultations (647.26 euros).

**Figure 2:**
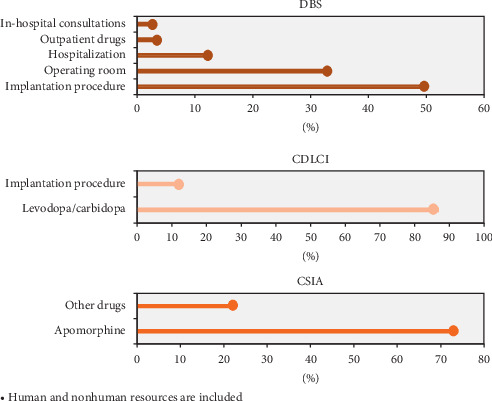
Resources used according to treatment. Human and nonhuman resources are included.

The average annual cost per patient in successive years after implantation was 1,295 euros (standard deviation of 1,395 euros). Thirty-nine percent of this amount corresponded to outpatient pharmaceutical products (505.05 euros), 34% to in-hospital consultations (440.30 euros), 9% to hospitalization (116.55 euros), and 4% to emergency room care (51.80 euros) (Figure 2). For the year when the device battery was replaced, the average annual cost per patient was 16,969 euros (standard deviation of 2,055 euros).  Table 2 shows the average cost of treating a patient receiving DBS over a five-year period.

### 4.2. Continuous Duodenal Levodopa/Carbidopa Infusion (CDLCI)

Twenty-seven patients received CDLCI treatment from 2016 to 2018. The average cost over the three years was 50,554 euros (standard deviation of 39,074 euros) per patient. Seventy-two percent of the cost was for in-hospital drugs (36,398.88 euros), 12% was for hospitalization (6,066.48 euros), and 6% was for outpatient pharmaceutical products (3,033.24 euros).

The average annual cost of infusion pump implantation was 23,212 euros (13,000 euros standard deviation), and it was determined that levodopa/carbidopa accounted for between 82 and 93% of the cost of the entire treatment.  Table 3 shows the details for the period from 2016 to 2018. The average accumulated cost over three years was 125,783 euros (standard deviation of 1,639 euros), and 107,748 euros corresponded to the cost of levodopa/carbidopa.

The average annual cost over the years following implantation of the infusion pump was 41,190 euros (standard deviation of 2,118 euros).

Table 4 shows the five-year costs of patients treated with levodopa/carbidopa, allowing comparisons of the cost of the disease for patients treated with the different therapies.

### 4.3. Continuous Subcutaneous Apomorphine Infusion (CSAI)

At the organization in which this study was conducted, 18 patients received CSAI treatment during the period from 2016 to 2018. The average cost per patient accumulated over three years was 29,943 euros (standard deviation of 17,643 euros). Most of the patients received a combination of treatments, although one of the patients was under exclusive CSAI therapy during the period from 2016 to 2018 (Table 5). The average annual cost was 34,118 euros (standard deviation of 2,459 euros), with 74% of this cost (25,090 euros) corresponding to apomorphine and 23% of the cost to the consumption of other complementary drugs (Figure 2). The total cost for this patient from 2016 to 2018 was 102,355 euros.

Table 6 shows the costs at 3 and 5 years for patients treated with apomorphine, allowing comparisons of the cost of the disease.

### 4.4. Comparative Analysis

**Figure 3:**
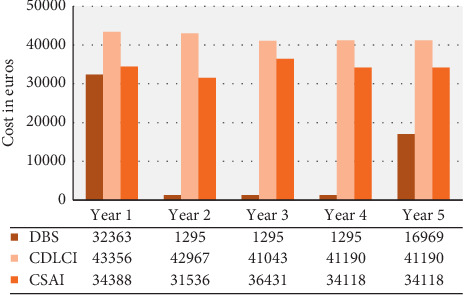
Cost comparison between DBS, CDLCI, and CSAI over five years. ^*∗*^Years 4 and 5 are based on estimated costs.

Figure 3 shows the cost comparison per patient over a 5-year period for each type of treatment.

## 5. Discussion

A review of the most recent literature in this field revealed studies that support the methodology we used [8–10]. Analysing costs using RWD is relevant because a prevalence is evaluated throughout the study period. Such analyses are performed once diagnosis has been established and all direct costs have been identified. Likewise, when patients have a long survival time, retrospective studies, such as the present study, are highly suitable. This is consistent with other studies that have used the same approach [9].

Other research has shown the high costs of PD. Rodríguez-Blázquez et al. analysed 70 articles related to this topic, gathering information from countries such as Belgium, Finland, and the United States and showed that healthcare costs represent the greatest burden and increase as the disease progresses. Similarly, it has been observed that social costs are lower in the early stages when the autonomy of the patient is preserved [9, 10].

As found in the analysis of each treatment, DBS has a high initial cost due to associated procedures and controls, although the costs of the adjuvant drugs over the subsequent years are lower. According to our results, the first year accounts for 60% of the total cost of the five years of treatment (Table 2), whereas other studies have found that this figure is only 32.2%. These differences may be due to the difference in methodologies and resources considered in the analysis [10, 11]. In the United States, it has been shown that DBS costs USD $37,481 per patient during the first year, which is similar to the figure obtained in the present study. Likewise, in Germany, it has been found that the cost decreases markedly after the first year, costing 2,689 euros per patient [9]. In our study, it was found that after an initial investment of 32,363 euros, the cost over the following years was 1,295 euros.

Compared to research conducted in Germany and England based on a healthcare provider approach that sought to compare the same three therapies, we observed that CDLCI had a higher cost than the other therapies. The least expensive treatment was DBS, with the previous study reporting similar figures to those found in this study [12].

A previous study showed that the direct cost of CSAI amounted to 104,500 euros, whereas the present study showed that it costs 170,591 euros. The cost of apomorphine alone, excluding other drugs that the patient received as adjunctive treatments, accounted for between 71% and 73% of the total cost of the treatment. In the articles reviewed, the costs of the drugs ranged from 490 to 2,960 euros per patient per year, which may have increased as the symptoms of PD worsened.

The results obtained are in line with the available literature. In 2013, Valldeoriola et al. carried out a five-year comparative analysis of healthcare costs associated with the same three therapeutic options. However, they estimated costs via questionnaires of experts and noted that the mean cumulative 5-year cost per patient was significantly lower for DBS (88,014 euros) than for CSAI (141,393 euros) or CDLCI (233,986 euros).

The data analysed in the present study are accurate, as the study was based on RWD. Likewise, the literature has shown that the differences in results between various studies are due to specific contextual factors and the features of each health system. Costs increase at advanced stages of the disease, and refractoriness causes less effectiveness in controlling symptoms and, in many cases, abandonment or discontinuation of treatment [13, 14]. In a retrospective study using a bottom-up methodology, the cost increased with each year of the disease and in some periods doubled from USD $4,317 to USD $9,658. It is important to note these variations between the different studies to ensure more precise and comparable designs. [15].

As costs vary by country according to each health system's characteristics and the availability of resources [15], economic assessments have become an integral part of decision-making for clinicians and public policy makers [13]. Information on costs is needed not only from a general standpoint but also to ensure that the aggregation of data from different sources can improve the efficiency of the systems [13, 16].

The limitations of most studies involve incomplete descriptions of the methods used, the source of information, the subjectivity of some measures in the results, and different timelines and methodologies [11]. The main limitation of the study carried out in this IHO was the small number of individuals included. Furthermore, this study was designed according to patients' traceability. Therefore, certain inputs that cannot be traced or assigned at the individual level were not included, although these costs can be deemed minimal.

## 6. Conclusions

The analysis enabled us to ascertain the details of the costs incurred by treatment with DBS, CDLCI, and CSAI and compare them to the results found in the literature on the subject. DBS is one of the least expensive treatments over a five-year period. For DBS, the largest costs are incurred when the implantation procedure is performed and when the battery is changed. The costs of CDLCI and CSAI are similar to those observed in Spain in the study by Valderiola et al. Compared with studies from other countries, our results are different but provided mostly similar findings.

During the review of the literature and comparisons of the results, we found that it is important to take into account the nuances that can be drawn from the study framework. PD has a high cost burden from all perspectives (patient, payer, provider, and social) that increases as the disease progresses. Due to current trends that have led to an increase in life expectancy, greater survival rates, and an increase in the disease period of PD, it is imperative to understand and clarify the costs of treating advanced PD with second-line therapies.

Ascertaining the cost per patient over a complete cycle or period of care enables the determination of the cost incurred by patients for each type of intervention. Furthermore, it allows an understanding of the relationship between the results and the resources necessary to obtain such results. For this reason, a cost-per-patient system provides relevant information for decision-making about alternative forms of treatment.

The reviewed studies on costs associated with this pathology applied different methodologies and addressed the issue from different approaches. Some did not specify whether the patients were in an early or advanced stage, which caused significant variations in costs significant and made costs more difficult to compare. This study highlights the types of treatment analysed and describes how the data obtained from the patients included in the study were exploited.

Currently, measuring the concept of “value” in healthcare, which includes maximizing outcomes that are important to people at the lowest possible cost, is increasingly seen as being the key for transforming healthcare delivery towards more efficient and sustainable models. The challenge involves implementing value-based healthcare (VBHC) in practice, both at the clinical and organizational level, and aligning this effort with integrated care and population health management perspectives. It is necessary to improve methodologies of cost analysis [17] and transform data into powerful patient-level information. However, there is a far greater focus on measuring health outcomes than measuring costs. Therefore, this research intended to fill this gap by providing a comparative RWD cost analysis of second-line therapies for patients with PD.

## Figures and Tables

**Table 1:** Patients treated with DBS from 2016 to 2018.

Year	2016	2017	2018
Neurostimulator device implantation	16 patients	19 patients	18 patients
Battery replacement	0 patients	5 patients	2 patients

**Table 2:** Total cost of DBS over five years of treatment.

Year	Year 1 (implantation)	Year 2	Year 3	Year 4	Year 5 (battery change)	Total cost
Average annual cost	€32,363	€1,295	€1,295	€1,295	€16,969	€53,217

**Table 3:** Average annual cost of CDLCI from 2016 to 2018.

Year	2016	2017	2018
Average annual cost	€43,356	€42,967	€41,043
Levodopa/carbidopa average annual cost	€35,368	€39,917	€38,211
% of total cost related to levodopa/carbidopa	82%	93%	93%

**Table 4:** Total cost of CDLCI over five years of treatment.

Year	Years 1–3	Year 4	Year 5	Total cost
Average cost	€125,783	€41,190	€41,190	€208,163

**Table 5:** Cost of CSAI for one patient.

Year	2016	2017	2018
Average annual cost	€34,388	€31,536	€36,431
Apomorphine average annual cost	€25,191	€22,459	€27,619
% of total cost related to apomorphine	73%	71%	76%

**Table 6:** Total cost of CSAI over five years of treatment.

Year	Years 1–3	Year 4	Year 5	Total cost
Average annual cost	€102,355	€34,118	€34,118	€170,591

## Data Availability

The data are not public but available on request to the research team.
